# Microfluidics for 3D Cell and Tissue Cultures: Microfabricative and Ethical Aspects Updates

**DOI:** 10.3390/cells11101699

**Published:** 2022-05-20

**Authors:** Tania Limongi, Francesco Guzzi, Elvira Parrotta, Patrizio Candeloro, Stefania Scalise, Valeria Lucchino, Francesco Gentile, Luca Tirinato, Maria Laura Coluccio, Bruno Torre, Marco Allione, Monica Marini, Francesca Susa, Enzo Di Fabrizio, Giovanni Cuda, Gerardo Perozziello

**Affiliations:** 1 Department of Applied Science and Technology, Politecnico di Torino, Corso Duca degli Abruzzi 24, 10129 Turin, Italy; tania.limongi@polito.it (T.L.); bruno.torre@polito.it (B.T.); marco.allione@polito.it (M.A.); monica.marini@polito.it (M.M.); francesca.susa@polito.it (F.S.); enzo.difabrizio@polito.it (E.D.F.); 2 Nanotechnology Research Centre, BioNEM Laboratory, Department of Experimental and Clinical Medicine, University Magna Graecia of Catanzaro, 88100 Catanzaro, Italy; francescoguzzi@aol.it (F.G.); patrizio.candeloro@unicz.it (P.C.); francesco.gentile77@gmail.com (F.G.); tirinato@unicz.it (L.T.); coluccio@unicz.it (M.L.C.); 3 Department of Medical and Surgical Sciences, University Magna Graecia of Catanzaro, 88100 Catanzaro, Italy; parrotta@unicz.it; 4 Research Centre for Advanced Biochemistry and Molecular Biology, Department of Experimental and Clinical Medicine, University Magna Graecia, 88100 Catanzaro, Italy; stefania.scalise@unicz.it (S.S.); valeria.lucchino@unicz.it (V.L.); cuda@unicz.it (G.C.)

**Keywords:** 3D cell cultures, microfluidics, lab on chip, in vitro cell cultures, 3Rs principles

## Abstract

The necessity to improve in vitro cell screening assays is becoming ever more important. Pharmaceutical companies, research laboratories and hospitals require technologies that help to speed up conventional screening and therapeutic procedures to produce more data in a short time in a realistic and reliable manner. The design of new solutions for test biomaterials and active molecules is one of the urgent problems of preclinical screening and the limited correlation between in vitro and in vivo data remains one of the major issues. The establishment of the most suitable in vitro model provides reduction in times, costs and, last but not least, in the number of animal experiments as recommended by the 3Rs (replace, reduce, refine) ethical guiding principles for testing involving animals. Although two-dimensional (2D) traditional cell screening assays are generally cheap and practical to manage, they have strong limitations, as cells, within the transition from the three-dimensional (3D) in vivo to the 2D in vitro growth conditions, do not properly mimic the real morphologies and physiology of their native tissues. In the study of human pathologies, especially, animal experiments provide data closer to what happens in the target organ or apparatus, but they imply slow and costly procedures and they generally do not fully accomplish the 3Rs recommendations, i.e., the amount of laboratory animals and the stress that they undergo must be minimized. Microfluidic devices seem to offer different advantages in relation to the mentioned issues. This review aims to describe the critical issues connected with the conventional cells culture and screening procedures, showing what happens in the in vivo physiological micro and nano environment also from a physical point of view. During the discussion, some microfluidic tools and their components are described to explain how these devices can circumvent the actual limitations described in the introduction.

## 1. Introduction

Traditional cell cultures are performed in two-dimensional (2D) systems such as Petri dishes, multiwell plates or flasks. However, they cannot realistically mimic the morphophysiological complexity of the original three-dimensional (3D) in vivo environment from which the cells of specific lines originate [1]. Without opposing animal experimentation but promoting its responsible application, the development of alternative cell culture systems tries to ensure compliance with the 3R principles. Reduction (reduction in the animals used for in vivo tests), Refinement (experimental design optimization to limit stress and affliction to laboratory animals) and Replacement (total or partial replacement of animal testing with alternative valid methods) are increasingly desired and strongly recommended as fundamental ethical aspects in the use of animals in scientific experiments [2].

Three-dimensional cell cultures can better mimic in vivo conditions than two-dimensional monolayer cell cultures, since, after isolation, cells generally lose their original morphology, changing the way they perform most of their physiological functions. Growth on an adhesion substrate results in cellular loss of polarity and it understandably influences intracellular trafficking, the functionality of subcellular compartments and some functions such as cell signaling and secretion, limiting the access to the culture media’s nutrients, the gaseous exchanges and the removal of waste substances [3]. In 2D cell cultures the complex network of regulatory interactions in the extracellular matrix (ECM), cells and tissue are altered, therefore the use of properly designed 3D culture systems assists researchers in obtaining more reliable results, deepening our understanding of what really happens in vivo [4,5]. Many studies report data concerning the significant differences in the morphology, protein expression, differentiation, viability, and functionality of cells grown in 2D or 3D systems [1,3,6,7,8,9,10,11].

Three-dimensional cell cultures can be successfully used for many different applications, including cell or drug screenings [12,13,14,15] and tissue generation (engineering) purposes [16,17,18]; however, the reproduction of a biomimetic environment is challenging [19,20,21]. It is very important to replicate as close as possible the original in vivo physiological cell microenvironment. When dealing with 3D cell cultures, one of the big issues is to provide a physiological exchange of substances (gas and molecules) between cells and their related microenvironment, inward for the cell nutrients and outward for the waste products. It is known that unfortunately 2D cell culture usually results in low nutrients and/or hypoxic regions related to cellular aggregation phenomena, biological media and gas consumption rates [22].

The use of optimized ECM-analog biomaterials with physico-chemical and structural properties, able to guarantee optimized degradation or residence rate and micro/nanoporosity, improves in vitro cell proliferation, differentiation, and interactions [23,24].

Decellularized engineered ECM and bioreactor-based solutions constitute valid alternatives to 2D cell cultures. The application of decellularization protocols in tissue engineering and regenerative medicine limits the possible immune response in the transplanted host by removing all the potential immunogenic biomaterials. The non-immunogenic ECM can be re-cellularized with autologous or stem cells, carrying out a fully personalized medicine approach [25,26]. In addition, micro-bioreactors can be regarded as a major step toward more complex organ-on-a-chip (OoC) systems [27], providing manageable 3D cell culture settings usually including suitable fluid flow supply and low amounts of chemicals and cells [28,29,30,31].

In the next sections, we critically treat the major issues related to the exchange of substances between blood and cells, detailing the passive mechanisms of transport on which these phenomena rely. In this context, we report how these mechanisms are physiologically reproduced in vitro, comparing biomolecules exchanges in 2D cell culture and 3D microfluidic devices. Some microfluidic tools and their components are described to explain how these devices can support the research, optimizing in vitro tests in a more reproducible, effective, and ethical way.

## 2. Discussion

During the last few decades, the use of in vitro systems has presented as solid alternative to animal experimentation and allowed the implementation of cellular and subcellular experimental models, moving the interest of the scientific community towards the “ever smaller”. Starting from a theoretical background that takes into consideration the in vivo substances’ physiological exchange and the theory behind the movement of particles across a capillary’s membrane, in the next subsections we will describe how 3D cell scaffolds and, in particular 3D microfluidics cell culture solutions, could replace traditional 2D cell cultures, reducing the sacrifice of laboratory animals in scientific research, in agreement with the 3Rs principles of the European Union.

### 2.1. Physiological Exchange of Substances

In physiological conditions, the exchange of substances and gases between cells and the environment takes place thanks to blood microcirculation at the level of the capillaries. Blood circulates from the arterioles to capillaries, then to venules and the topology of these vessels changes according to the different tissues that are sprinkled. Some beds are structured as trees, others as arcades or sinuses or portal systems [32]. The capillary density (CD) depends on the varying oxygen and nutrients requirements to keep a stable metabolism. The average CD in human tissue is around 600 per mm^3^ and it changes according to the different organism’s tissues. The CD is higher in the brain, kidneys, liver and myocardium (around 2500–3000 per mm^3^), reduced in the phasic units of the skeletal musculature (around 300–400 per mm^3^) and even lower in the bones, fat, connective tissues and in the tonic units of the skeletal musculature (less than 100 per mm^3^) [33].

Considering an average capillary diameter of 8 μm and length of 5 mm [34], we can calculate the average distance between adjacent capillaries which is around 30–40 μm (around 1–3 cell width). To reach a particular cell, molecules exit the capillary and cross one or two cells to reach the target one. A capillary vessel can be considered as a tube consisting of a single endothelial cells’ layer less than 1 μm thick [35]. There are three types of capillaries: (i) the continuous type with cells tightly joined together, which are present in muscles, nerves, and connective tissues; (ii) the fenestrated type, with cells so thin that internal vesicles form small pores 100 nm thick and 6 nm in diameter (typically around 1000 pores/μm^2^); (iii) the discontinuous type with distinct intercellular gaps (around 5 μm in diameter) and a broken basement membrane, commonly found in organs such as the liver, spleen, and bone marrow, the functions of which include the injection or extraction of whole cells, large molecules and extraneous particles in/from the blood stream [36].

**Figure 1:**
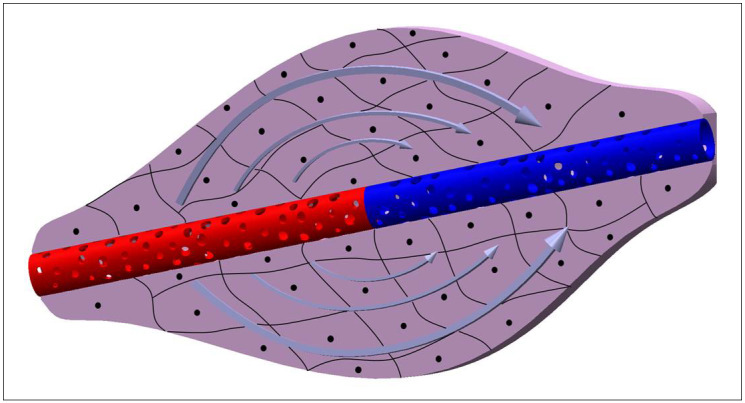
The scheme represents an arterial capillary (in red) connected to a venous capillary (in blue) and surrounded by a generic tissue constituted by cells. The arrows show the movement of fluids around the capillary, due to filtration (in the arterial side) and reabsorption (in the venous side).

The nutritive and waste substances pass the capillary pores by means of a dynamic equilibrium established between the hydraulic pressure and the osmotic pressure gradients between the blood inside the capillaries and the interstitial fluid in the ECM. In particular, the blood’s osmotic pressure (oncotic pressure) is around 25–30 mmHg and it is higher than the one of interstitial fluid which is around 0 mmHg. The osmotic pressure gradient is constant between the blood circuit and the surrounding tissues including the arterial capillaries and the venous capillaries. While the hydraulic pressure in blood decreases, going from the arterial capillaries (where it is around 40 mmHg) to the venous capillaries (which is around 15 mmHg), in the interstitial fluid it is around 2 mmHg. Since, in the arterial capillaries, the hydraulic pressure in the blood is higher than the oncotic pressure, filtration, a flow that goes from capillaries to tissues, occurs. On the contrary, in the venous capillaries, the oncotic pressure is higher than the hydraulic one and liquids are reabsorbed in capillaries due to a flow from tissues to capillaries (Figure 1).

The exchange of molecules between blood microcirculation and cells forming tissues and organs is due to filtration, reabsorption and at the same time diffusion through the capillary membrane of substances at a different concentration on the two sides of the capillary membrane. The presence of capillaries drastically reduces the diffusion length, since they are very close to each other.

### 2.2. Theory behind the Molecule Transport Mechanisms

Referring to the theory behind the movement of particles across a capillary’s membrane, it can be considered a unidimensional motion, assuming the concentration gradient across the membrane as constant. This approximation is certainly valid in the dynamic environment of the biological systems where, while cells consume nutrients and produce wastes, capillaries provide nutrients and remove wastes, keeping the concentration gradients across capillary’s membrane constant.

The flux of molecules due to diffusion can be calculated as:

(1) $$J_{dM}=-P\Delta C,$$

where

- $\Delta C=\left(C_{2}-C_{1}\right)$ is the concentration gradient of a generic molecule between the external and internal part of the capillary membrane;
- P is the permeability coefficient and can be calculated as:

(2) $$P=\frac{D_{M}\alpha }{\Delta x}=\frac{D_{M}n\pi R^{2}}{\Delta x},$$

where

- Δx is the capillary membrane thickness;
- α is the partition coefficient and can be calculated as:

(3) $$\alpha =\frac{N\pi R^{2}}{A}=n\pi R^{2},$$

where

- N is the number of pores;
- A is the capillary surface;
- R is the pore radius;
- n is the pore density;
- $D_{M}$ is the membrane diffusion coefficient and can be calculated as:

(4) $$D_{M}=\epsilon D,$$

where

- ϵ is the hindrance coefficient and it depends on the particle and membrane pore dimension and the trajectory of the particle within the pore and can be calculated as:

(5) $$\epsilon =\epsilon_{1}\epsilon_{2}={\left(1-\frac{r}{R}\right)}^{2}\epsilon_{2},$$

where

- $\epsilon_{2}$ is a coefficient that depends on the trajectory of the particle inside the pore;
- r is the particle radius (it is an approximation which considers the molecules passing the pore to have a spherical shape);
- D is the diffusion coefficient which can be calculated as:

(6) $$D=\frac{kT}{6\pi \eta r},$$

where

- k is the Boltzman constant;
- T is the temperature;
- η is the blood viscosity;

This last equation is valid if the particle which diffuses has a spherical shape. In this work, the particles will be considered, as first approximation, to have a spherical shape.

While the flux of molecules through the capillaries can be calculated as a function of the pressure and the osmotic gradient across the capillary.

(7) $$J_{fM}=-\alpha \frac{C_{1}+C_{2}}{2}\varepsilon L_{p}\left(\Delta p-\Delta \pi \right),$$

where

- $\Delta p=\left(p_{2}-p_{1}\right)$ is the hydraulic pressure gradient across the capillary membrane;
- $\Delta \pi =\left(\pi_{2}-\pi_{1}\right)$ is the osmotic pressure gradient across the capillary membrane;
- $L_{p}$ is the filtration coefficient and can be calculated as:

(8) $$L_{p}=\frac{n\pi R^{4}}{8\eta \Delta x},$$

For instance, considering a pore density of 100 pore/μm^2^ [37], a pore diameter of 6 nm, a capillary thickness of 1000 nm, at a body temperature of 37 °C, a glucose molecule, with a relative radius of 4.5 Å and present at a concentration of 80 mg/dl in blood, will diffuse through the capillary at 28.5∙10^−2^ mg/m^2^s (roughly 7.6∙10^17^ molecules/(m^2^s)). In a capillary with 8 μm of diameter and 1 mm of length, there will be a flux of glucose due to the diffusion of 5.7∙10^−28^ mg/s (roughly 1.5∙10^−9^ molecules/s). In the same conditions, considering a pressure gradient of 40 mmHg and an osmotic pressure gradient of 25 mmHg, the flux of glucose due to filtration will be of 3.6∙10^−6^ mg/m^2^s (roughly 9.6∙10^12^ molecules/m^2^s). In a single capillary, the filtrated glucose will be 7.2∙10^−33^ mg/s, corresponding to roughly 1.9∙10^−14^ molecules/s. Thus, in the case of glucose, the dominant phenomenon is diffusion. Considering a total number of capillaries in the human body equal to 4∙10^9^, it can be calculated a movement of glucose equal to different kilograms per day. In general, the exchange of substances between blood and tissues is dominated by diffusion, referring to a very small space (as mentioned before: 2–3 cell width, around 40 μm).

### 2.3. Cell Microenvironment: Static and 3D Cell Screening

Mimicking the best possible cellular microenvironment does not only mean having control overflows, since many parameters such as shear stress, cell interactions, pH, CO_2_, temperature, and O_2_ variations affect its regulation and balance. Although it is well-known that in any kind of cell screening applications, it is very important to control the cell microenvironment, the current in vitro systems are still far from having an appreciable level of control on it [38]. Generally, supports such as Petri dishes, flasks and vials are used to culture cells in a static condition, leading to temperature and chemical gradients that could make it difficult to maintain homeostasis [39]. In addition, the use of standard static cell culture supports requires a lot of manual procedures, such as the addition of fresh culture medium and the removal of the old one, resulting in time-demanding procedures for the operator and stressful conditions for cells.

One of the alternatives to static cell culture procedures is the use of in vivo experiments that are undoubtedly able to reduce the gap between in vitro and in vivo screening procedures. Unfortunately, in vivo experimentation in basic and pre-clinical practice involves a considerable waste of resources, both in monetary and ethical terms, considering the number of animals to be sacrificed. Over the years and with the progress in biomedical and technological fields, there has been a tendency to drastically reduce in vivo experiments using the advanced alternatives to animal testing towards the 3Rs (Replacement, Reduction, Refinement) approach. [40,41,42]. Although replacing should be the main purpose of the 3Rs, its implementation in the short-term is ambitious, while minimizing the number of animals and refining their welfare should be feasible in the short/middle-term [43].

A solid alternative to animal tests is cell scaffolds, as 3D cell culture can effectively mimic the cellular and tissue microarchitecture [44,45]. Both for pharmacological screening and pathologies modelling, 3D scaffolds represent one of the most successful platforms for biomedical applications [46,47,48,49].

**Figure 2:**
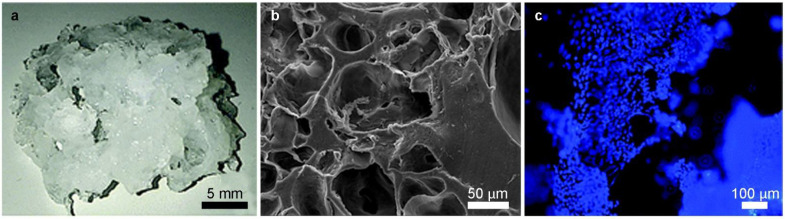
3D PVA scaffolds in which stem cells are grown [50]. (**a**) macroscopic view of the 3D wet scaffold at room temperature; (**b**) scanning electron microscope cross sectional details of the 3D structure; (**c**) fluorescence microscopy image of DAPI stained cell homogeneously distributed on a Matrigel coated PVA scaffold.

Dattola et al. developed a poly(vinyl) alcohol (PVA) 3D scaffold where stem cells grew and differentiated into cardiac cells (Figure 2) [50]. These scaffolds mimicked the mechanical properties of ECM in which cardiomyocytes proliferated in vivo, demonstrated by the contractile property detected in the cardiomyocytes grown on the proposed scaffold. However, it was found that cells colonized only the outermost part of the scaffold, since they could not survive deep into the bulk volume, because the nutrients were not properly provided in the innermost layers of the 3D scaffolds.

They provided a continuous supply of nutrients and oxygen while removing metabolic wastes by creating an artificial network. This enabled the production of large, engineered tissues and the assembly of multiples organoids or spheroids to generate a whole system in vitro. Microfluidic systems also allowed precise culture conditions and better monitoring of cells. Once cells were cultured three-dimensionally in vitro, these considerations should be taken in account to reproduce in vivo conditions. In this contest microfluidic scaffolds effectively tried to solve the main issues related to the establishment of 3D cell cultures. Microfluidic systems, as an amelioration of the 3D scaffold methods, aimed to reduce in vitro cultured cells’ discomfort and death related to inadequate nutrients distribution and catabolites clearance. Microfluidic cell culture solutions allowed non-invasively time-saving sampling and screening, reducing post-seeding inhomogeneity, since their tunable design and networks enable multiple and automated procedures [51].

Microfluidics assisted 3D cell culture by mechanically and chemically controlling cellular microenvironment, gas and temperature gradients, shear-stress and most of the relevant physical-chemical properties. Nowadays, these solutions are customized for the main biomedical applications, including cell therapy, drug, and toxicity assays (Table 1).

In Table 1, we summarized some of the most representative and recent 3D microfluidic cell culture applications found in the literature from 2015. Most of these studies concern drug screening and OoC applications, witnessing the increasing interest in regenerative and personalized medicine. Consulting the papers cited in the table, it is possible to extrapolate how, in general, microfluidics can reduce time and costs, allowing the implementation of high-throughput screening in drug discovery and disease models.

In the work of Shin et al. [56], a reproducible protocol to induce intestinal morphogenesis in microfluidic platforms using Caco-2 cell line was reported. Authors established a disease model, developing in vitro intestinal epithelial layers suitable to study intestinal physiology and host-microbiome interactions. Regional differentiation markers such as KRT20, villin, CEACAM1 and CYP3A4 were considerably expressed in the villus region, suggesting cytodifferentiation of the 3D epithelial layers.

Bircsak et al. [64] used an OrganoPlate LiverTox™ platform to co-culture three different cell lines: (i) iPSC-derived hepatocytes (ii) THP-1 monoblast and (iii) endothelial cells, respectively, in the ratio of 5:5:1, reproducing a hepatic model for hepatotoxicity. The liver model was evaluated for albumin, urea, alpha-fetoprotein synthesis, cell viability and CYP3A4 activity over 15 days. A total of 159 hepatotoxic compounds were screened, evaluating liver response to drugs using viability, nuclear size, urea and albumin assays.

In recent years, devices such as “body-on-a-chip” or “human-on-a-chip” have become ever more common and some of the recently proposed systems are already have the ability to reproduce multi-organ interactions. In Maschmeyer et al. [68], the authors introduced a four-organ-chip system modeling human intestine, liver, skin and kidney. The device, composed by two polycarbonate cover-plates and by a PDMS-glass chip, can accommodate both a blood and an excretory system, each controlled by a dedicated peristaltic micro-pump. This device has been designed to support absorption, distribution, metabolism and excretion (ADME) and profiling of substances, along with repeated toxicity testing of drugs. Authors successfully co-cultured the different cell types for 28 days, reporting a high cell viability and discrete physiological tissue architecture over the entire period. Finally, metabolic and gene analysis confirmed the establishment of a reproducible homeostasis between all four tissues.

### 2.4. Microfluidic Cell Screening Devices

**Figure 3:**
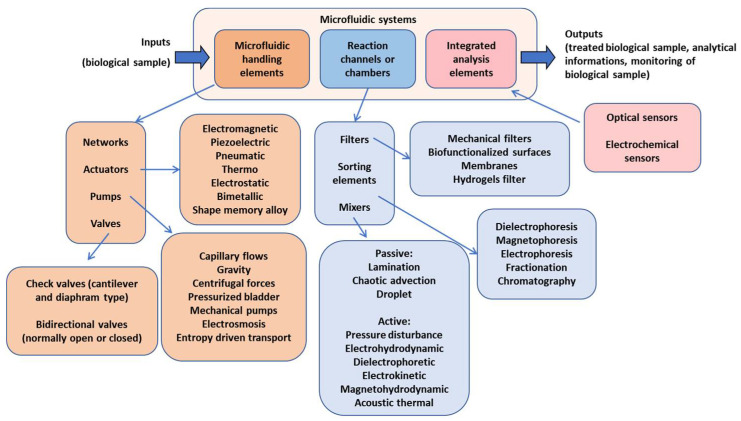
Scheme reporting some of the main components which can constitute a microfluidic device.

Microfluidics allows an accurate local control and is thus able to provide a biologically relevant and well-defined cellular microenvironment. It also allows us to manage bioanalysis at high resolution and with greater precision than conventional technologies, enhancing and improving in vitro cellular imaging and tests [82,83]. In Figure 3, some of the components that a microfluidic device can integrate are summarized in a logic scheme, since the functional integration and interconnections of all its components can be quite complex [20,84,85,86,87]. The relative lack of integration of microfluidics, more generally of micro and nanotechnology in biological laboratories, may be due, in part, to the bridging of the gap between the engineers who design and manufacture the devices and the biomedical users who would ultimately use them. This decoupling has often led to device prototyping immaturity from an application point of view, and is not easy to use and not totally reliable in regard to the reproducibility of the data collected with their use.

**Figure 4:**
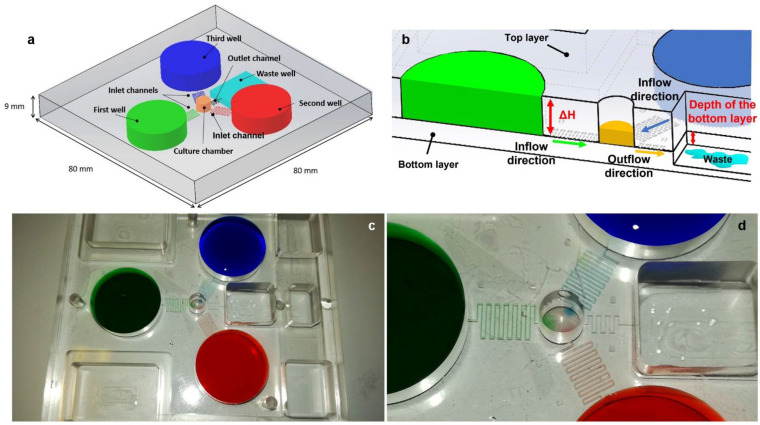
Passive microfluidic platform for cell culturing [92]. (**a**) isometric sketch of the microfluidic device; (**b**) zoom in of the sketch showing the physical principle to drive the liquids inside the device; (**c**,**d**) picture and picture’s details of the device showing how the different liquids diffuse in the culture chamber.

Among many authors designing and testing microfluidic platforms to simplify and optimize protocols for biological experiments [88,89,90,91], in the following we reported in detail some of the most recently microfabricated solutions developed by our research group. In Guzzi et al. [92], we developed a microfluidic platform able to culture cells in a semi-static environment. Cells were maintained inside a 3 mm deep well where culture medium (~60 μL) can circulate below and above cells trying to reproduce the microcirculation. The device is composed of three different cylindrical reservoirs (each one filled with ~2 mL of liquids) arranged at 120° around the smaller central culture chamber, with a lower height compared to the reservoirs. A waste well collected waste liquids coming from the culture chamber (Figure 4a). The major advantage of this device is its passivity, since it did not include any actuation to move liquids, but they are simply driven by gravity in a special interconnected system of communicating wells. Height differences generated a pressure gradient moving fluids from the reservoirs to the central chamber and, finally, to the waste well manufactured onto a deeper layer (Figure 4b). Fresh culture media mixed each other in the central chamber feeding the cells with new nutrients. Mixing in the central chamber mainly occurred by diffusion for the low flow rate of the system (~6.2 μL/h for each inlet channel) and to the laminar flow of microfluidics (Figure 4c,d). This device, designed both for adherent and non-adherent cells, can be maintained in a miniaturized or in a traditional incubator to constantly monitor the cell growth, using an inverted optical microscope and physiological parameters such as the pH and the dissolved oxygen variation. Furthermore, it was an open device, so users can access each area, loading of withdrawing samples and reagents using a conventional pipette.

**Figure 5:**
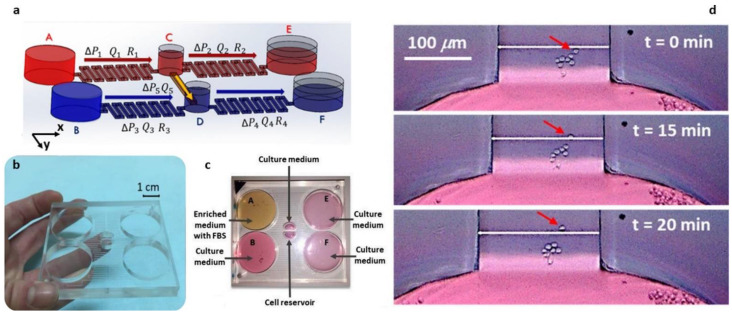
Microfluidic device for chemotaxis studies. (**a**) isometric sketch of the microfluidic circuits showing the working principle of the device; (**b**,**c**) images of the device; (**d**) time lapse images of a chemotaxis experiment.

Coluccio et al. [93] reported the development of a passive device composed of two parallel microfluidic networks connected via a transversal channel (Figure 5a–c). Different fluids’ volume in the reservoirs a and e, and the reservoirs b and f generated a flow rate in the parallel channels. In detail, reservoirs were filled to reach the same hydrostatic pressure in reservoirs A and B, C and D, E and F, so pressure gradients ΔP1, ΔP 2, ΔP3 and ΔP4 were equal. The hydraulic resistances (R1, R2, R3 and R4) in the channels of the device were regulated to have the flow rates Q1 = Q2 and Q3 = Q4. In addition, the same volumes of liquid in the central reservoirs C and D (ΔP5 = 0), meaning the same hydrostatic pressure, assured a flow rate Q5 = 0. Therefore, there was no flow in the transverse channel. Maintaining the different concentration of substances in the parallel channels, it was possible to create a concentration gradient in the transverse channel, in which chemotaxis experiments were performed. Authors used suspension cells (Jurkat cells) to demonstrate a chemotactic process based on the attraction of cells towards a medium containing Fetal Bovine Serum with an average speed of about 0.36 μm/min, comparable with other conventional devices used to perform similar experiments (Figure 5d) [55]. Potentially, the surfaces of the microchambers and microchannels of these devices can be further functionalized with different synthetic or biological moieties to increase their biocompatibility or to target a specific application [94,95,96].

## 3. Conclusions

The constant need of living cells to acquire nutrients and oxygen and to eliminate waste substances for the maintenance of their physiological processes requires increasing efforts to solve the lack of cell culture protocols. One of the possible solutions lies in the design of new systems based on the optimization of convective flow to ameliorate cell growth and tissue vasculogenesis [97]. In contrast to methods undertaken in the early years when physicists and engineers started to develop the first prototypes of lab-on-chip as mere platforms for diagnostic applications [98,99,100], nowadays we can observe how microfluidics can be actively integrated in both research and clinical biomedical fields.

Microfluidic devices for cell culture and tests provide new dynamic in vitro microenvironments and methods to observe and modulate cellular responses to biological or pharmacological stimuli. Only by increasing the number of microfluidic devices for cell biology developed to address current obstacles, such as biomimetic potential and reproducibility, can the unique strengths of these devices become ever more accessible to the whole biomedical community as common daily laboratory tools.

Modern microfluidics solutions and lab-on-chip systems enable the construction of 3D cellular culture and co-cultures and realistic mimicking of the in vivo tissue-level microenvironments, including pathological inflammatory/cancer states [101,102,103]. Innovative and effective 3D cell culture microfluidic models have started to be proposed more frequently as solid alternatives for tissue regeneration and drug screening applications [104]. The possibility to cultivate, screen and analyze cells directly isolated from patients paves the way to clinical treatments that perfectly suit the patient, switching from the traditional “one size fits all” approach to a personalized one [105]. Moreover, automated methods will increase the reliability of procedures that will become more precise and faster without the need for large quantities of samples [106]. Microfabricated microfluidic 3D cell culture technologies highly impact preclinical-to-clinical translation, both in the pharmaceutical and regenerative medicine contexts, mimicking, in some cases, human physiology better than the more traditional in vitro models. The recognition of the value of the microfluidic models by collecting and interpreting data from different sources, reinforced by the support of regulatory agencies, will be able to ensure an effective in vitro-to-in vivo translation through quantitative model systems and pharmacology application. The integration of these increasingly precise and reproducible devices, together with the possibility of analyzing a large number of experimental variables derived, at the same time, from the same sample or from different ones, will allow us to mimic increasingly complex systems from both an anatomical and functional point of view, considerably reducing the number of animals sacrificed for in vivo experiments. An active communication between bioengineers, physics, clinics and regulators could successfully assist the transition towards the more ethical limited and optimized use of the animal experiment. The prompt development of effective microfluidic 3D models may successfully reduce the sacrifice of laboratory animals in scientific research, in accordance with the 3Rs regulation of the European Union, since a valid compromise must be established between preserving animal welfare whilst conducting high-quality and ethical science.

## Figures and Tables

**Table 1:** Summary of a selection of the most representative and recent 3D microfluidic cell culture applications.

Microfluidic Platform Type	Application	Cell Lines	References
Resin 3D-printed system (VeroClear, MED610 resins)	Cell Culture, LC-MS/MS single cell analysis	BPAECs (Bovine Pulmonary Artery Endothelial Cells), MDCK (Madin-Darby Canine Kidney)	[52]
Microwell-based PDMS-membrane-PDMS sandwich multilayer chips	Spheroid formation, OoC	C3A (liver)	[53]
Two-stage temperature-controlling system used to generate decellularized extracellular matrix (dECM) hydrogel microspheres	dECM hydrogels microsphere formation, cell culture	Schwann cells (nervous tissue), PC12 (adrenal gland)	[54]
Injection-molded Polystyrene array	OoC, angiogenesis	HUVEC (Human Umbilical Vein Endothelial Cells), fibroblasts	[55]
PDMS-gut-on-a-chip device either with a straight channel or a non-linear convoluted channel, transwell-embedded hybrid chip	OoC	Caco-2 (colon)	[56]
Cyclo-olefin-polymer (COP) transparent bioreactor	On-chip platelet production	imMKCLs (immortalized MegaKaryocyte progenitor Cell Lines)	[57]
PDMS soft lithography replicas of superficial channels 3D-printed in different resins (Clear, Model, Tough, Amber, Dental resins)	OoC	HUVEC (Human Umbilical Vein Endothelial Cells), fibroblasts	[58]
PDMS bone-mimicking extracellular matrix composite device	Angiogenesis, OoC	SW620 (colon), MKN74 (stomach)	[59]
Single-chamber commercial microfluidic device	OoC, disease model, drug screening	Primary human hepatocytes, EA.hy926 (human endothelial), U937 (pleural effusion), LX-2 (hepatic stellate cell)	[60]
Collagen scaffold	OoC	Caco-2 (colon)	[61]
Cellulose-based device	Chemotaxis, invasion assay	A549 (lung)	[62]
Polymerized High Internal Phase Emulsion (polyHIPE) system	OoC	hES-MPs (human Embrionic Stem cell-derived Mesenchymal Progenitor cells)	[17]
OrganoPlate LiverTox™	Drug screening, OoC	Induced pluripotent stem cell (iPSC)-derived hepatocytes (iHep), endothelial cells, THP-1 monoblast (peripheral blood)	[63]
Injection-molded Polystyrene array	Drug screening	HeLa (uterus, cervix), NK-92 (peripheral blood)	[64]
Resin 3D-printed system (VeroClear)	Spheroid formation	OSCC (Oral Squamous Cell Carcinoma), HepG2 (liver)	[65]
3D-printed device	Circulating Tumour Cells (CTCs) isolation	MCF-7 (breast), SW480 (colon), PC3 (prostate), 293T (kidney)	[66]
PDMS-based device	Spheroid formation, disease model, drug screening, OoC	Rat primary hepatocytes, HSCs (Hepatic Stellate Cells)	[67]
PDMS-glass chip and Polycarbonate cover-plates	Four OoC	EpiIntestinal™, HepaRG (liver), HHStec (Human primary Hepatic Stellate cells), RPTEC/TERT-1 (human proximal tubule)	[68]
PDMS-based device	OoC	Hepatocytes from primary and iPS-derived cells	[69]
Three-layered glass device	OoC, disease model, drug screening	Primary human hepatocytes, LSECs (Liver Sinusoidal Endothelial Cells), Kupffer cells (liver)	[70]
Three-layered glass device	OoC, disease model, drug screening	Primary human hepatocytes, iPSC (induced-Pluripotent Stem Cells), endothelial cells, Kupffer cells (liver)	[71]
Silicon scaffold fabricated by deep reactive ion etching	OoC, disease model, drug screening	PHH (Primary Human Hepatocyte), non-parenchymal cells	[72]
PDMS “open-top” device	Angiogenesis, spheroid formation	HDMEC (Human Dermal Micro-vascular Endothelial Cells), Primary human lung fibroblasts, U87MG (nervous tissue)	[73]
PDMS based device	Angiogenesis, OoC	hLFs (human Lung Fibroblasts), HUVECs (Human Umbilical Vein Endothelial Cells)	[74]
Two-layered glass-PDMS hybrid system	Spheroid formation, invasion assay, drug screening	U87 (nervous tissue)	[75]
3D-printed system (Vero White Plus FullCure 835 resin)	Angiogenesis, cell culture, drug screening	bEnd.3 (mouse brain endothelial cell line)	[76]
Double-casting of PDMS, with master mold made of PMMA.	Spheroid formation, drug screening	Caco-2 (Colon), NHDF (Normal Human Dermal Fibroblast), HepG2 (liver), A549 (lung)	[77]
3D-hydrogel device	Drug screening, OoC	hCMEC/D3 (endothelial cell), HUVECs (Human Umbilical Vein Endothelial Cells), primary neurons, astrocytes	[78]
PDMS based device	OoC, drug screening	C3A (liver), EA.hy926 (endothelial)	[79]
PMMA-PDMS hybrid system and bioprinted hydrogel scaffold	OoC, angiogenesis	HUVECs (Human Umbilical Vein Endothelial Cells), neonatal rate cardiomyocytes	[80]
PDMS based device	OoC, disease model, drug screening	hiPSCs (human induced Pluripotent Stem Cells), CMs (Cardiomyocytes) differentiated from hiPSCs	[81]
